# Circular Optical Phased Arrays with Radial Nano-Antennas

**DOI:** 10.3390/nano12111938

**Published:** 2022-06-06

**Authors:** Qiankun Liu, Daniel Benedikovic, Tom Smy, Ahmad Atieh, Pavel Cheben, Winnie N. Ye

**Affiliations:** 1 Department of Electronics, Carleton University, Ottawa, ON K1S 5B6, Canada; vincentliu3@cunet.carleton.ca (Q.L.); tomsmy@cunet.carleton.ca (T.S.); 2 Department of Multimedia and Information-Communication Technologies, University of Zilina, 010 26 Zilina, Slovakia; 3 University Science Park, University of Zilina, 010 26 Zilina, Slovakia; 4 Optiwave Systems, Inc., Ottawa, ON K2E 8A7, Canada; ahmad.atieh@optiwave.com; 5 National Research Council Canada, Ottawa, ON K1A 0R6, Canada; pavel.cheben@nrc-cnrc.gc.ca

**Keywords:** silicon photonics, silicon-on-insulator platform, optical phased arrays, grating nano-antennas, far-field pattern, steering range, field-of-view, light detection and ranging

## Abstract

On-chip optical phased arrays (OPAs) are the enabling technology for diverse applications, ranging from optical interconnects to metrology and light detection and ranging (LIDAR). To meet the required performance demands, OPAs need to achieve a narrow beam width and wide-angle steering, along with efficient sidelobe suppression. A typical OPA configuration consists of either one-dimensional (1D) linear or two-dimensional (2D) rectangular arrays. However, the presence of grating sidelobes from these array configurations in the far-field pattern limits the aliasing-free beam steering, when the antenna element spacing is larger than half of a wavelength. In this work, we provide numerical analysis for 2D circular OPAs with radially arranged nano-antennas. The circular array geometry is shown to effectively suppress the grating lobes, expand the range for beam steering and obtain narrower beamwidths, while increasing element spacing to about 10 μm. To allow for high coupling efficiency, we propose the use of a central circular grating coupler to feed the designed circular OPA. Leveraging radially positioned nano-antennas and an efficient central grating coupler, our design can yield an aliasing-free azimuthal field of view (FOV) of 360°, while the elevation angle FOV is limited by the far-field beamwidth of the nano-antenna element and its array arrangement. With a main-to-sidelobe contrast ratio of 10 dB, a 110-element OPA offers an elevation FOV of 5° and an angular beamwidth of 1.14°, while an 870-element array provides an elevation FOV up to 20° with an angular beamwidth of 0.35°. Our analysis suggests that the performance of the circular OPAs can be further improved by integrating more elements, achieving larger aliasing-free FOV and narrower beamwidths. Our proposed design paves a new way for the development of on-chip OPAs with large 2D beam steering and high resolutions in communications and LIDAR systems.

## 1. Introduction

Integrated optical phased arrays (OPAs) are enabling technologies for efficient generation and fast scanning of optical beams, yet eliminating the need for mechanical movements. On-chip OPAs are particularly appealing due to their compact size, lowered power consumption, and improved beam steering capabilities [1,2]. Non-mechanical OPAs are the key building blocks in many nanophotonic applications. This includes communications [3,4], mapping and metrology [5], and light detection and ranging (LIDAR) [6], to name a few. For LIDARs, with applications in autonomous vehicles, satellites, or drone navigation, OPAs with narrow beam widths and wide-angle steering are often required. While narrow beams can be tailored by integrating a large set of OPA antennas, wide-angle steering is obtained through minimizing the element size and array spacing, the latter preferably at a distance less than half of the operating wavelength to suppress sidelobes [1]. However, such a small element spacing is challenging to achieve for optical communication wavelengths (1.26–1.65 μm). For the element spacing larger than a half of the wavelength, grating sidelobes in the far-field rise substantially, limiting the aliasing-free beam steering range. 

To date, integrated OPAs typically use one-dimensional (1D) linear arrays [7,8,9,10,11,12,13] and two-dimensional (2D) planar arrays with either rectangular [14,15,16,17,18,19,20] or circular [21,22,23,24,25,26] arrangements. For 1D arrays, wavelength-dependent grating nano-antennas are typically used for beam steering. On the other hand, rectangular 2D arrays rely on nano-antennas with phase shifters to achieve beam steering. In silicon waveguides, the minimum element spacing can be reduced to about one optical wavelength (~1550 nm at telecom frequencies) for 1D linear OPAs [7,8,9,10,11,12,13]. This results in a maximum steering range of about 90° for azimuthal and elevation angles. The wavelength-tuned steering is typically within a ±10° range, limited by the antenna bandwidth. Moreover, a small element spacing creates a large angular beamwidth for the main beam. To achieve a high OPA resolution, a small angular beamwidth is required. In 2D rectangular arrays, the minimum element spacing can be a multiple of the operating wavelength. Still, the element separation remains restricted by the size of the nano-antenna element [14,15,16,17,18,19,20]. Although rectangular arrays yield a higher resolution, the steering range is lower compared to 1D arrays. Typically, there is a trade-off between the field of view (FOV) and the resolution, which proves to be challenging to address simultaneously. 

Circular OPAs are promising alternatives to 1D linear and 2D rectangular arrays, where a circular array can offer a large steering range and high resolution concurrently [21,22,23,24,25,26]. For circular OPAs, the high resolution and wide scanning can be obtained with efficient sidelobe suppression. However, to date, feeding systems of 2D circular OPAs remain inefficient.

In this work, we report on a novel 2D OPA design with a circular configuration. We show that the circular antenna array effectively suppresses the grating lobes, while simultaneously providing a large steering range and narrow beamwidth. For this, we numerically investigated two different circular arrays arranged radially. Compared to the other circular arrangements [21,22,23,24,25,26], our proposed OPAs are fed by a central circular grating coupler with a high coupling efficiency. Leveraging radially positioned nano-antennas and an efficient central grating coupler, the circular OPAs can yield an aliasing-free azimuthal FOV of 360°. The elevation angle FOV remains limited by the far-field beamwidth of the antenna and array configuration, which scales with the number of the OPA elements. For a sidelobe suppression of 10 dB, a 110-element OPA offered an elevation FOV of 5° and an angular beamwidth of 1.14°, while an 870-element array provides an improved elevation FOV of 20°, with an angular beamwidth as small as 0.35°. The proposed circular OPAs integrated on a silicon-on-insulator (SOI) platforms feature large aliasing-free FOVs and narrow beamwidths of the reference beam simultaneously. Our analysis suggests that the performance of the circular OPAs can be further improved by integrating more elements.

## 2. Circular Optical Phased Array

**Figure 1:**
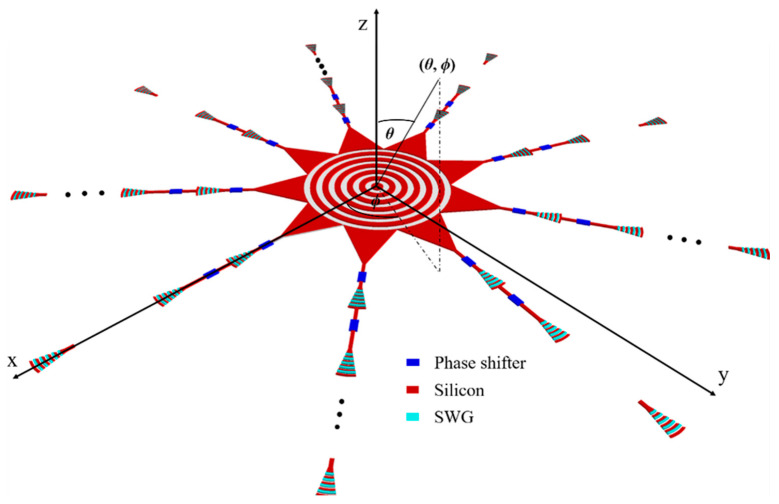
Schematic view of a circular optical phased array with radially arranged nano-antennas. Here, the SWGs are the subwavelength grating engineered nano-antennas, and *θ* and *ϕ* are elevation and azimuthal angles.

The schematic view of the proposed circular OPA with radially arranged nano-antennas is shown in Figure 1. The circular OPA is designed for an SOI platform with a 220 nm thick silicon on top of a 2 μm buried oxide (BOX) layer. The upper cladding is 2 μm thick silicon dioxide. The refractive indexes of Si and SiO_2_ are 3.476 and 1.444, respectively, at a wavelength of 1550 nm [27]. The OPA components are optimized for the transverse electrical (TE) polarization. The overall OPA is fed through an input optical interface comprising a central circular grating coupler followed by adiabatic tapers. Adiabatic tapers provide a smooth transition between the central grating coupler and output waveguides. The output waveguides are connected by phase shifters and individual nano-antennas. The respective OPA elements are described below.

### 2.1. Central Grating Coupler

**Figure 2:**
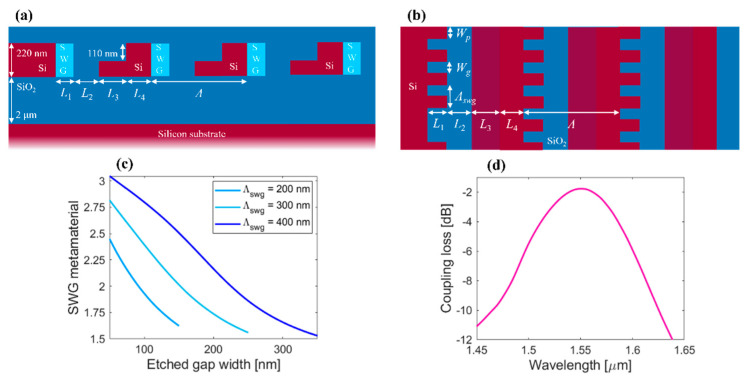
2D schematics of the central grating coupler with L-shaped SWG-engineered radiating segments in (**a**) side view and (**b**) top view. (**c**) The effective index of the SWG waveguide (*n*_swg_) as a function of the etched gap width (*W*_g_) for different subwavelength grating periods (*Λ*_swg_) of 200 nm, 300 nm, and 400 nm. (**d**) Calculated fiber-chip coupling efficiency of the central grating coupler as a function of the wavelength.

The OPA is fed by a central circular grating coupler, ensuring a compact footprint of the OPA. The input circular grating coupler has a maximum outer radius of 15 μm, corresponding to the coupler length. Grating coupler simulations were performed by using a commercial 2D finite difference time domain (FDTD) solver by Optiwave to optimize its structural and performance parameters. The performance of the circular grating coupler was engineered to match the upward radiation beam profile to the Gaussian-like optical fiber mode. The grating coupler was designed using an L-shaped waveguide geometry [28] with subwavelength grating (SWG) metamaterial sections to reduce back-reflections [29,30]. The SWGs are non-resonant nanophotonic structures with pillar segmentation with dimensions much smaller than the wavelength of light, suppressing reflection and diffraction effects [31]. Over the years of research, SWG metamaterials have captured a strong interest due to their eminent potential of customizing optical and light propagation properties in planar silicon waveguides [31,32,33]. The *L*-shaped grating exploits vertically asymmetric scatters to maximize the light coupled upwards, while minimizing the light radiated towards the Si substrate. The 2D schematics (side and top views) of the input grating coupler are shown in Figure 2a,b, respectively. Each grating period comprises four segments with longitudinal dimensions *L*_1_, *L*_2_, *L*_3_, and *L*_4_. Here, *L*_1_ and *L*_2_ are deep-etch grating trenches with metamaterial index *n*_swg_ and SiO_2_, while *L*_3_ and *L*_4_ are lengths of 110 nm shallow-etch trench and unetched Si segment, respectively. The coupling efficiency is estimated as a product of the power fraction radiated upwards and an overlap between the radiated field and the optical fiber mode. In the present work, a standard optical fiber (SMF-28) is used, following a mode field diameter of 10.4 μm with 1/e^2^ intensity beam width, at a reference wavelength of 1550 nm. The design was optimized with a genetic algorithm [34,35], where lower and upper boundaries for grating parameters were set as follows: [*L*_1_ = *L*_2_ = *L*_3_ = *L*_4_ = 100 nm, and *n*_swg_ = 2.0] and [*L*_1_ = *L*_2_ = *L*_3_ = *L*_4_ = 300 nm, and *n*_swg_ = 3.0]. The variation of the peak radiation angle was set to less than 3° to maintain a vertical coupling. As a result, the grating coupler has an upward radiation of 86.3% and overlap with fiber mode of 76%. The overall coupling efficiency achieved was 66% (−1.83 dB loss), using optimized grating coupler parameters: [*L*_1_ = 240 nm; *L*_2_ = 144 nm; *L*_3_ = 208 nm; *L*_4_ = 129 nm; and *n*_swg_ = 2.4]. Figure 2d shows the calculated coupling efficiency of a central grating coupler as a function of the wavelength.

### 2.2. Adiabatic Taper

An adiabatic taper was optimized for a smooth transition between central grating coupler and output Si waveguides. The taper length between a 500 nm wide Si waveguide and a 8.45 μm wide output waveguide is determined by modal analysis. A 15 μm long adiabatic taper provides an input-to-output transmission in excess of 94% according to the 3D FDTD simulations.

### 2.3. Grating Nano-Antenna

**Figure 3:**
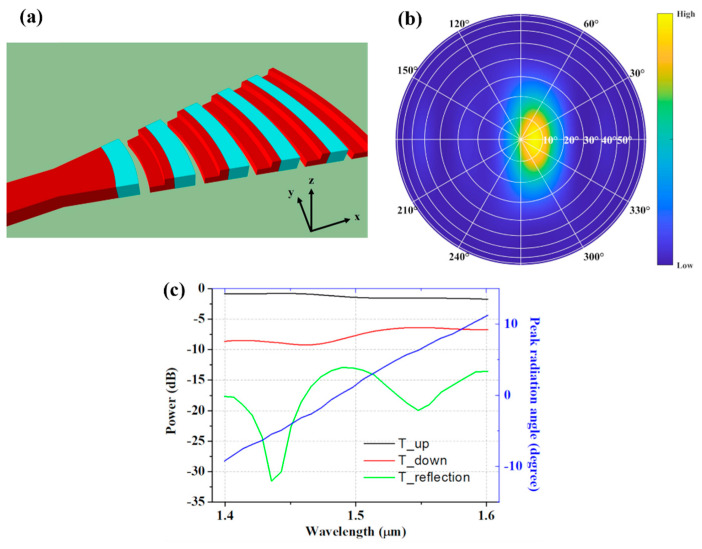
(**a**) 3D schematic of the nano-antenna using Si (red) and the SWG-engineered metamaterial (blue). (**b**) The far-field radiation pattern of the nano-antenna at a wavelength of 1550 nm. (**c**) 3D FDTD simulation of the nano-antenna radiated power (left axis) and peak radiation angle (right axis) as a function of the wavelength.

Surface-emitting antennas are the key elements in OPA systems. The OPA steering range is determined by the far-field pattern of the individual nano-antenna. The system steering range with respect to the elevation angle can be increased by exploiting the wavelength dependence of the radiation angle of the grating antenna. To take advantage of the large maximum theoretical FOV of a circular array, broadband nano-antennas are needed. In this work, we advantageously adopted an *L*-shaped configuration [29,30], similar to the elements discussed in Section 2.1. A 3D schematics of the nano-antenna is shown in Figure 3a. 

This high-directionality nano-antenna [36] has a metamaterial transition to reduce back-reflections at the waveguide-to-antenna interface and is compatible with silicon photonic foundry manufacturing [28,37]. The designed nano-antenna provides broadband operation, with a less than 1 dB penalty on a radiation power in the wavelength range from 1400 nm to 1600 nm. The nano-antenna is comprised of five periods and each period contains four segments. Segment lengths are: [*L*_1_ = 280 nm, *L*_2_ = 130 nm, *L*_3_ = 196 nm, and *L*_4_ = 134 nm]. The first grating trench is a subwavelength grating (SWG) engineered nanostructure with a refractive index of 2.32. The grating lines have a circular curvature with a wedge opening angle of 26°. The nano-antenna has a compact footprint of only 5.5 μm by 2.5 μm (length by width). The far-field radiation pattern of the nano-antenna, emitting at a reference wavelength of 1550 nm, is shown in Figure 3b. Here, the white concentric circles correspond to the elevation angle *θ* from 0° (surface normal) to 90°, while the straight lines represent the azimuthal angle *ϕ* from 0° to 360°. The peak radiation angle of the nano-antenna is defined as the point of maximum radiation efficiency, i.e., maximum power that is radiated upwards. For a wavelength of 1550 nm, the maximum radiation efficiency corresponds to angles *θ*_0_ = 6° and *ϕ*_0_ = 0°, respectively. Figure 3c shows the full far-field radiation performance of the designed nano-antenna, including upward, downward, and back-reflected powers. In particular, the maximum radiation efficiency of -0.8 dB was obtained at 1440 nm, while its minimum of −1.73 dB was found at 1600 nm wavelength. Figure 3c (right axis, blue line) shows a peak radiation angle of the upward radiation as a function of wavelength. By varying the wavelength, we obtained a linear angle rotation of the upward radiation beam. The peak radiation angle changes from −9° at 1400 nm up to 11° at 1600 nm.

In the proposed circular OPA, each ring includes nano-antennas with equal angular spacing of 2*π*/*M* between two adjacent antennas within a respective concentric ring. The input power coupled through the central grating into individual outputs is 1/*M*. The nano-antennas are radially arranged with respect to the OPA center. The minimum radius of the central ring is *R*_0_, while the radius increment between two consecutive rings is *dr*. In the proposed OPA, there are *N* concentric rings and *M* antennas in each ring. The overall OPA consists of *M* × *N* elements, having an aperture size of 2*π*[*R*_0_ + *dr*(*N* − 1)]. The OPA power feeding to individual antennas can be realized via independent coupling or a cascaded geometry. 

## 3. Results and Discussion

### 3.1. Beam Forming in Circular Array

In our circular OPA, the orientation of the individual antennas within the same ring is varied with respect to the angular spacing between the adjacent elements. This yields a rotation of radiation pattern with an angle of *α*_m_ along the azimuthal direction. The array far-field (AFF) is calculated as [38]:

(1) $$AFF\left(\theta ,\phi \right)\;=\sum_{n\;=\;1}^{N}\sum_{m\;=\;1}^{M}\alpha_{mn}e^{-i\frac{2\pi }{\lambda }\sin\left(\theta \right)\cos\left(\phi -\alpha_{m}\right)R_{n}}M\left(\nu ,\alpha_{m}\right)P_{0}\left(\theta ,\phi \right)\;$$

where the element weight coefficient is $\alpha_{mn}=1/\sqrt{MN}$, *λ* is the wavelength, *R*_n_ is the radius of the respective ring, *P*_0_ is the far-field radiation pattern of nano-antenna along *x*-axis with zero rotation angle, and *θ* and *ϕ* are elevation and azimuth angles, respectively. *M* is the rotation matrix with a rotation angle of *α*_m_, and the vector ν = (r=1,θ=0). The rotation angle $\alpha_{m}=2\pi \left(m-1\right)/M$ is the angle between *m*^th^ antenna and the *x*-axis.

**Figure 4:**
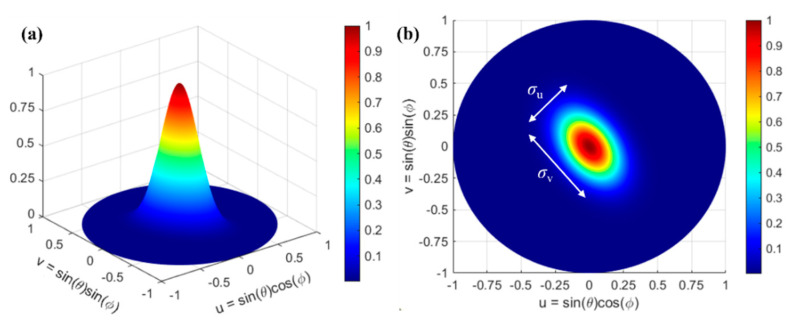
The generated Gaussian beam of the *m*^th^ nano-antenna with *σ*_u_ = 0.1 and *σ*_v_ = 0.18. (**a**) 3D view and (**b**) 2D view.

We simulated the performance of the circular OPA, considering the sidelobe suppression, angular beamwidth, and steering range. To investigate the beam formation and beam steering, the radiation pattern of the nano-antenna was emulated by a 2D elliptical Gaussian beam. The nano-antenna has a vertical light emission with a peak upward radiation angle of 0° of the 2D Gaussian beam [39]:

(2) $$\begin{array}{c} g\left(u,\nu \right)=A\exp\left(-\left(a{\left(u-u_{0}\right)}^{2}+2b\left(u-u_{0}\right)\left(\nu -\nu_{0}\right)+c{\left(\nu -\nu_{0}\right)}^{2}\right)\right)\\ a=\frac{\cos^{2}\alpha_{m}}{2\sigma_{u}^{2}}+\frac{\sin^{2}\alpha_{m}}{2\sigma_{\nu }^{2}}\\ b=\frac{\sin2\alpha_{m}}{4\sigma_{u}^{2}}-\frac{\sin2\alpha_{m}}{4\sigma_{\nu }^{2}}\\ c=\frac{\sin^{2}\alpha_{m}}{2\sigma_{u}^{2}}+\frac{\cos^{2}\alpha_{m}}{2\sigma_{\nu }^{2}} \end{array}$$

where *A* is the amplitude of the Gaussian beam, u=sin(θ)cos(ϕ), ν=sin(θ)sin(ϕ), and $\left(u_{0},\nu_{0}\right)$ is the center of the Gaussian beam. *σ*_u_ and *σ*_v_ denote the full width at half maximum (FWHM) of the Gaussian along the *u* and *ν* axes. The calculated FWHM of our nano-antenna is *σ*_u_ = 0.1 and *σ*_v_ = 0.18, respectively. The generated Gaussian beam, with a normalized amplitude *A* = 1, of the *m*^th^ nano-antenna is shown in Figure 4.

The AFF of circular OPA is determined by four parameters, namely *N*, *M*, *R*_0_, and *dr*. To systematically study the performance of the circular OPA, we calculated the sidelobe suppression and angular beamwidth for varying input parameters (*N*, *M*, *R*_0_, and *dr*). The sidelobe suppression is defined as the difference between the peak of the main lobe (normalized to 0 dB) and the maximum of the sidelobes within the full scanning range. The angular beamwidth is defined as the FWHM of the main lobe. The circular OPA was optimized via genetic algorithm [34,35] to tailor the sidelobe suppression at the steering angle (*θ*_0_ = 0°, *ϕ*_0_ = 0°), at which all nano-antennas are in phase. For each optimization step, we set an upper boundary of total element number to 110, a lower boundary for the radius increments between adjacent rings to 8 μm, with the minimum distance between adjacent nano-antennas on the ring larger than *dr*.

**Figure 5:**
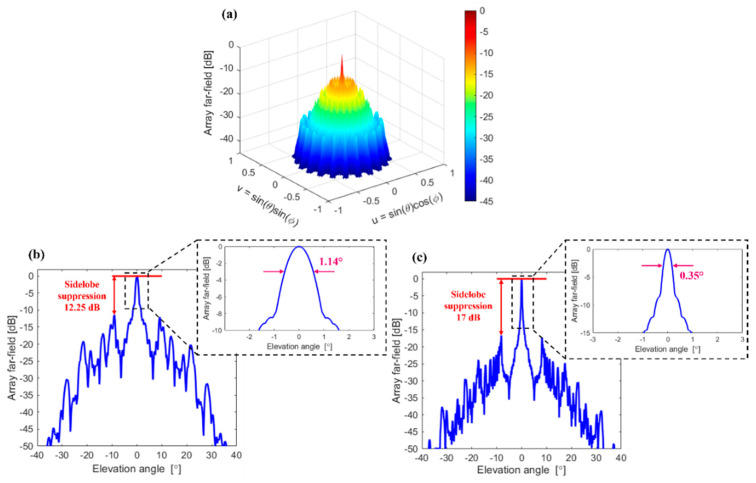
(**a**) Simulated far field of a radially configured circular OPA with parameters: *N* = 10, *M* = 11, *R*_0_ = 75 μm, and *dr* = 10 μm, for *θ*_0_ = 0°, *ϕ*_0_ = 0°. Array far field is shown in a logarithmic scale. Elevation cut of simulated array far field for (**b**) *N* = 10, *M* = 11, *R*_0_ = 75 μm, and *dr* = 10 μm and (**c**) *N* = 30, *M* = 29, *R*_0_ = 96 μm, and *dr* = 11 μm. Elevation cuts correspond to *θ*_0_ = 0°, *ϕ*_0_ = 0°. Insets of (**b**,**c**): enlarged views of main lobes of array far field for an elevation angle range −3° to 3°.

As a result, the optimized array comprises 110 elements, with *N* = 10, *M* = 11, *R*_0_ = 75 μm, and *dr* = 10 μm. The designed OPA configuration yielded a sidelobe suppression of about 12.2 dB. The simulated AFF in a logarithmic scale is shown in Figure 5a. The scanning region corresponds to a full hemisphere in a forward direction. For this study, a resolution of 1° was used in simulation (*θ* = 0°:1°:90° and *ϕ* = 0°:1°:360°). Figure 5b shows an elevation cut (at *ϕ* = 0°), from which the angular beamwidth of the circular OPA with radially arranged nano-antennas is determined. As shown in the inset of Figure 5b, the angular beamwidth is 1.14°. To correctly measure the angular beamwidth of the main lobe, the elevation cut was calculated with an angular resolution of 0.01° in the *θ* = −90° to *θ* = 90° range. The circular OPA with a radial nano-antenna configuration suppresses the grating lobes over the entire scanning range. The grating sidelobe suppression was obtained for an elevation angle *θ* varying from 0° to 90° and for azimuthal angle *ϕ* changing from 0° to 360°, respectively. Furthermore, the radial arrangement simplifies the layout implementation when compared to a conventional circular OPA with identical nano-antennas. The performance of the circular OPA depends on the total number of elements within the OPA. By increasing the number of OPA elements, we can obtain a higher sidelobes suppression as well as narrower angular beamwidths. By adjusting the optimization conditions and setting the total number of the element to 900, an optimized OPA design was found with the following parameters: *N* = 30, *M* = 29, *R*_0_ = 96, and *dr* = 11 μm. Figure 5c shows an elevation cut of the OPA with 870 elements. The larger OPA system has a sidelobe suppression of 17 dB and the angular beamwidth is reduced to 0.35°, as shown in the inset of Figure 5c.

### 3.2. Beam Steering in a Circular Array

**Figure 6:**
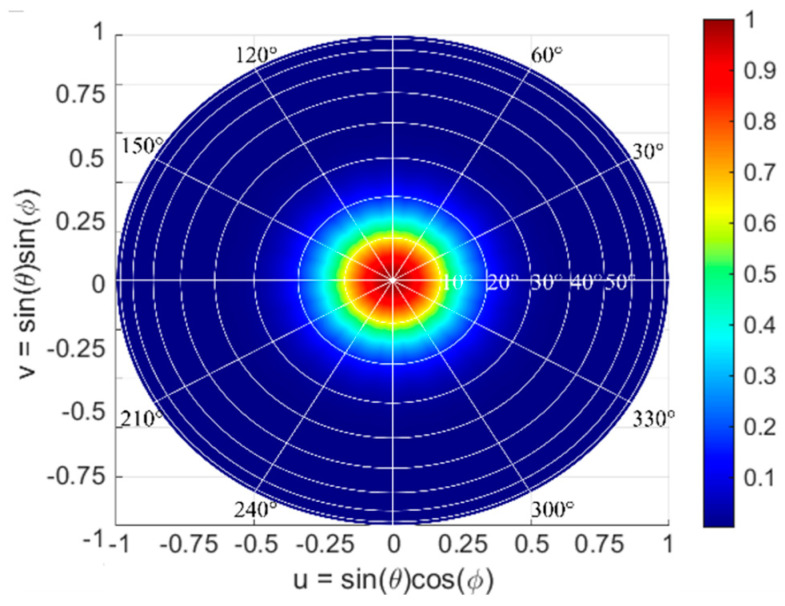
Far-field radiation diagram for a superposition of elliptical Gaussian beams, considering array configuration with *M* = 11 and *N* = 10.

The maximum steering range of the OPA determines the system field of view (FOV). The steering range is determined by the antenna bandwidth and is often limited by the grating lobes, particularly in 2D rectangular arrays. For the circular OPAs, the grating lobes are effectively suppressed, and the actual steering range is limited primarily only by the far-field pattern of the nano-antenna. Figure 6 shows the superposition of *M* rotated elliptical Gaussian beams. The dark blue area in Figure 6, where the elevation angle *θ* varies from 20° to 90° and azimuthal angle *ϕ* changes from 0° to 360°, denotes the region with zero radiation, which lies outside of the FOV of the circular array.

In the monochromatic operation, the steering range of the OPA system is limited to the area radiated into the far field defined by the nano-antenna radiation pattern. For instance, considering an input optical signal with a reference wavelength of 1550 nm, we can vary the steering angles (*θ*, *ϕ*) within the range defined by the radiation pattern of the nano-antenna. This variation can be performed by modifying the phase of each element through independently controlled phase shifters. 

**Figure 7:**
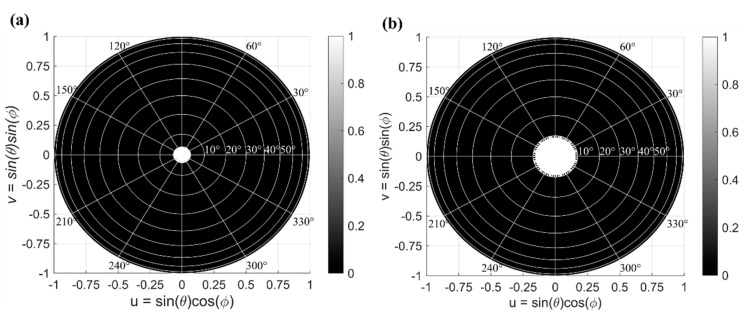
Steering range of the circular array with radially arranged nano-antennas for two design configurations: (**a**) 110 antenna elements with *N* = 10, *M* = 11, *R*_0_ = 75 μm, and *dr* = 10 μm and (**b**) 870 antenna elements with *N* = 30, *M* = 29, *R*_0_ = 96 μm, and *dr* = 11 μm. The white area represents the steering range.

To assess the steering range of the circular array, we estimated the sidelobe suppression of AFF. The AFF was calculated at different steering angles over the entire hemisphere. For the calculations, we considered *θ*_0_ = 0°:1°:20° and *ϕ*_0_ = 0°:1°:360°, while the resolution of 1° was used in simulations for *θ* from 0° to 90° and for *ϕ* from 0° to 360°. The effective steering range, which corresponds to the FOV, is defined as the set of all steerable angles (*θ*, *ϕ*). For our study, a minimum detectable contrast of 10 dB was set as the targeted sidelobe suppression. For instance, for a circular array design with *N* = 10, *M* = 11, *R*_0_ = 75 μm, and *dr* = 10 μm, steering at *θ*_0_ = 3° and *ϕ*_0_ = 60°, the calculated sidelobe suppression is 13 dB. For a circular array design with *N* = 30, *M* = 29, *R*_0_ = 96 μm, and *dr* = 11 μm, the sidelobe suppression level improves to 18 dB. To represent the steering range for OPAs, all steerable angles are projected on a *uν* plane. Figure 7 shows the top view of the steering range projected onto a hemisphere for considered OPA configurations, operating at a 1550 nm wavelength. Here, the bright areas correspond to the range of steerable angles, for which sidelobe suppression is larger than 10 dB. The bright area represents the actual FOV of the circular array with radially arranged nano-antennas, where the sidelobe suppression is larger 10 dB.

**Figure 8:**
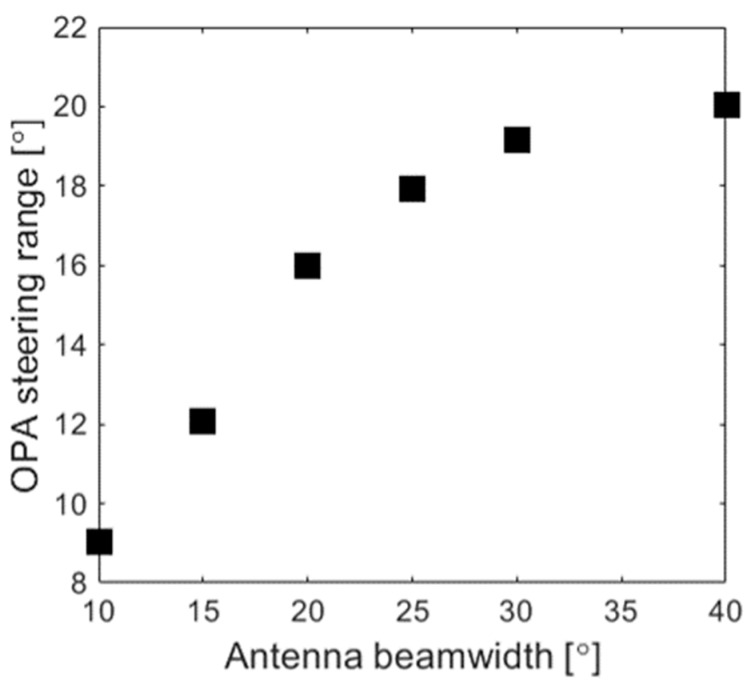
Simulated steering range (Δ*θ*) as a function of the antenna angular beamwidth in the elevation angle (*θ*) for an 870-element optical phase array.

In a radially arranged circular array, the beam steering can be performed with respect to the azimuthal angle *ϕ* over a wide range Δ*ϕ* = 360°. On the other hand, the limited steering range with respect to the elevation angle *θ* is a result of: (i) the restricted angular beamwidth in *θ* of the nano-antenna radiation pattern and (ii) the maximum sidelobe suppression level of the array steering at *θ*_0_ = 0° and *ϕ*_0_ = 0°. As observed in Figure 7, the circular array with 110 elements shows a steering range of Δ*θ* = 5° and *Δϕ* = 360°, while the larger array of 870 elements shows an improved steering range of *Δθ* = 10° and *Δϕ* = 360°. Given that the grating lobes are effectively suppressed, the main limitation for the achievable steering range in the elevation angle *θ* arises from a comparatively narrow angular beamwidth of the individual nano-antenna. This limitation is not fundamental to our circular OPA design and it can be overcome by using far-field broadened nano-antennas. The diffractive nano-antenna employed in this work has a beamwidth of 11°. Figure 8 shows the steering range Δ*θ* of the proposed array as a function of the nano-antenna angular beamwidth in the elevation angle *θ*. These data suggest that enlarging the nano-antenna beamwidth with respect to the elevation angle *θ* can directly improve the array steering range. In particular, for a circular OPA with 870 elements, improving the nano-antenna beamwidth with respect to *θ* to 20° yields a steering range Δ*θ* of 16°. It was also observed that the steering range Δ*θ* of the circular array saturates when the nano-antenna angular beamwidth becomes larger than 30°. This saturation effect on the steering range Δ*θ* can be mitigated by increasing the number of antennas, which also provides a larger sidelobe suppression ratio at *θ*_0_ = 0° and *ϕ*_0_ = 0°.

## 4. Conclusions

We proposed and numerically studied an on-chip circular OPA with radially arranged nano-antennas. The circular array is fed by an efficient central grating coupler based on *L*-shaped radiating elements. The use of a central grating coupler greatly facilitates the system’s compact layout and power distribution using short interconnecting waveguides. The circular OPA geometry effectively suppresses the grating lobes for an element spacing as large as ~10 μm. Leveraging the radially arranged nano-antennas, the circular OPA provides an aliasing-free azimuthal FOV from 0° to 360°, with a sidelobe suppression better than 10 dB. The array FOV in elevation angle is primarily limited by the angular beamwidth of the nano-antenna radiation pattern. The FOV in the elevation angle can be increased by scaling the number of array elements and by widening the antenna beamwidth. In summary, we have demonstrated the great potential of circular OPAs with radially arranged nano-antennas to enable large 2D beam steering with a high resolution. The circular OPA is a promising candidate for realizing practical LIDAR systems on a chip.

## Data Availability

Data are contained within the article.
